# Some Hints on the Preparation and Mounting of Microscopic Objects

**Published:** 1882-03

**Authors:** W. H. Walmsley


### SELECTIONS.

### SOME HINTS ON THE PREPARATION AND MOUNTING OF MICROSCOPIC OBJECTS.

BY W. H. WALMSLEY.

Our first paper of practical hints having been mainly devoted to balsam mountings, it was the writer's intention in the next (if ever written), to give other methods. But, on carefully reviewing the former, I find that directions have been given for mounting only very thin or flattened substances, with which but a small amount of balsam is needed between the slide and cover, and the latter lies parallel to the former, with but little inclination to tilt up on one side. There are, however, many objects so thick as to require some sort of a cell to contain the large amount of balsam necessary to cover them, and to prevent the excess from running out beneath the cover upon the slide; whilst other subjects are so delicate that the mere weight of the thinnest covering glass, pressing upon, would crush them out of all shape. We will, therefore, with the reader's permission, give some hints as to various methods of doing this before proceeding with other branches of our subject.

Undoubtedly, the most perfect cell that has yet been devised is one of glass. For very shallow cells, to be used in mounting diatoms, nothing can equal those cut from thin covering glass, such as are used exclusively by Moller in mounting his Typen- and Probe-Platten, and slides of arranged diatoms. These are cemented to the slides by a thin layer of balsam, heated to such a point as to expel all, or nearly all, of the turpentine; and nothing can excel the neatness and perfection of their appearance and finish, whilst they are, of course, quite permanent.

Where deeper cells are required, those cut from glass tubes of various diameters and shapes, or drilled from slips of different thicknesses, and cemented to the slides with marine glue, are to be highly recommended, as they are extremely neat in appearance, and entirely permanent in character. The objections to all glass cells are, the difficulty of preparing them by ordinary workers, and their extreme costliness, if purchased ready-made. If the latter consideration, however, be of no moment to the reader, I would say by all means use them, and nothing else, as they never give any trouble, and are always neat and handsome in appearance.

Block-tin makes a very good cell, which may be attached to the slide by marine glue the same as glass, and which will contain fluid balsam quite as well; but they must either be purchased of the dealers, or made with a special and expensive punch. They are more difficult to finish neatly than those of glass, and are only recommended by reason of their comparative cheapness. Brass curtain rings *may* be substituted for them, and if carefully handled in the mounting, may be found permanent and satisfactory; but my own experience has not been favorable toward the latter, either as to convenience of handling, permanency, or neatness of appearance.

Fortunately, we have a material admirably adapted to making cells, which any one can readily do for himself, and at a trifling cost. It is the ordinary white sheet-wax used in artificial flowers and which can be procured in three thicknesses, known as "ordinary," "double thick," and "pond lilly." A punch, specially constructed for cutting cells from these sheets, can be purchased from the opticians at the small cost of a dollar and a half; and enough to mount a dozen or more slides may be punched in a few moments.

A turn-table is necessary for attaching these cells to the slides, and some form of a self-centering one is recommended; the enhanced first cost being the only reason for hesitancy in making a choice. A simple form of *centering—not self*-centering, mind you—devised by myself, many years since, in my early microscopical days, and which costs but a fraction more than the simplest turn-table without it, has been found so satisfactory in my own work, as well as that of many friends who have adopted it, that I am led to describe it here. A sheet of thin brass, with a stop at the left hand end, precisely one and a half inches from the center, is attached to the surface of the whirling table by a small milled head, by which the distance of the edge of the brass plate from the table's center may be varied more than half an inch. A slide, precisely 3 x 1 inches, having been accurately centered on the table by means of the concentric rings turned about the center of the latter—and held in position by the usual spring clips—the guide plate is moved up until the lower left hand corner of the slide rests securely in the angle formed by the upper edge of the guide and the stop at its left; when the milled head is screwed up tight and the centering arrangement is ready to do its allotted work. It follows that any rectangular slide 3 x 1 inches, slipped under the spring clips and into the angle of the guide, must have *its* center exactly over that of the table, and a ring of cement once run upon it when so placed, can be followed by as many more as are desirable by simply replacing the slide in the same angle, without any trouble in adjusting. Since, however, very few slides are absolutely rectangular or have exactly parallel edges, it will be found convenient in practice to place a mark upon the left hand end of each before running the *first* layer of cement thereon, after which it may be replaced as often as desired, without loss of time. A writing diamond is the most convenient tool for doing this with, but a small scrap of paper pasted upon the slide will answer every practical purpose. The surface of the table should be sharply scratched at the angle formed by the guide-plate, so that in the event of having to move the latter, to accommodate a slide not originally ringed by it the centering adjustment may be returned to its original position in a moment and with absolute accuracy. My original device was cut from a piece of stiff cardboard, and secured to the table by means of the screws which attached the two spring-clips to the latter; and this primitive affair did excellent service for many a long year ere it gave way to my present truly *self*-centering instrument.

Let us take breath, for we have been a long while getting our turn-table; but, that invaluable instrument secured, at last we are ready for business. Taking a clean slide from our store box of same, we slip it between the clips of the table and bring it to a center at once, if we have followed the foregoing directions. First dusting its surface carefully with a camel's hair brush, we proceed to trace a flattened ring thereon, with balsam, diluted by chloroform to the consistency of *city* cream, using a small red sable brush for the purpose; ample directions for doing which were given in the preceding paper. The size of this ring must be the same as that of the cell we are about to place upon it, and the concentric lines turned upon the surface of the table, which are visible through the slide, furnishes us with an excellent guide to follow. Which of the three thicknesses of sheet wax we shall use, of course, will be determined by the thickness of the specimen to be mounted: if none (used singly) will furnish a cell sufficiently deep we must add one or more rings of the wax; taking care to coat each thoroughly with the balsam before adding the next ring. This may be all done without removing the slide from the turn-table, and thus a symmetrical and accurately centered cell may be built up in a very few minutes; and if necessary, may be at once filled with balsam and specimen, and the mounting completed. It is better, however, to set it aside for a day or two (in a dust-tight case) for hardening. And now, having completed our cell, be it of glass, metal, or wax, let us proceed to mount our specimen therein, without a legacy of air bubbles, or vacuoles; which is a very easy matter to do—when you once know how.

Suppose that our first specimen is a lot of foraminiferous shells of considerable size, which will require a cell of the depth made by a ring cut from a single thickness of pond-lily wax. Choosing such an one from our stock of already prepared cells—hold the slide very carefully over the lamp until it feels sensibly warm to the touch, then immediately fill the cell with pure limpid balsam, just sufficiently to reach the upper surface of same all around, and to form a slight convexity from side to side. The balsam may be squeezed from a collapsible tube, or lifted from the supply bottle by means of a glass rod, at pleasure; my own preference being decidedly for the former method, as being cleaner, more convenient, and keeping the balsam entirely free from dust, or evaporation. Now, gently warm the slide again to the same degree as before, and, with a clean needle, well heated over the lamp, explore the inner edges of the cell, down to its glass bottom, so, that if there be any vagrant air-bubbles imprisoned thereabouts, they may be freed, float to the surface and disappear of their own volition, or at the touch of a hot needle-point. A careful examination of the cell's contents should be made with a pocket lens, as a further surety that no imprisoned bubbles are left therein. And, it may be well to remark here, and for all the past as well as future operations, that they should be conducted as speedily as possible, since the balsam begins to harden as soon as brought into contact with the air, rendering it more and more difficult to manipulate the longer the operation lasts. The bell-glass should also be close at hand, to place over the slide during any pause in the work, to exclude dust.

The small shells—previously freed from all moisture or dust—may now be carefully dropped into the cell and moved into position with a warm, *not hot*, needle, until the desired amount is deposited therein. Examine again, to make sure no fugitive air-bubbles are left; and now we are ready for the covering-in. The glass circle for this purpose should be slightly smaller than the wax ring which forms the cell: thus, if the diameter of the latter be $\frac{3}{4}$ of an inch, the former should be $\frac{11}{16}$. The covers can be purchased in all sizes, one-sixteenth of an inch apart, from $\frac{1}{4}$ to 1 inch in diameter.

Taking up the cleaned circle with the forceps, and slightly warming it over the lamp, we proceed to apply it to the cell, precisely in the same manner as previously described for a balsam mount, in which no cell was employed; care being taken to let it fall gently so as to drive out the excess of balsam over the edge of the cell, and so down on to the surface of the slide, without moving the shells out of their positions or leaving any *vacuole*

within the cell. Should the latter mishap occur, the only remedy is to push the cover off to one side and apply sufficient fresh balsam to fill the vacancy; replacing the cover in position by a gentle sliding motion. This necessity, however, is to be avoided by all means, if possible, since it makes a "*smeary*" job, very difficult to clean off nicely afterward. Should any small air bubbles yet remain, after all our precautions, they need cause no annoyance or thought as they will disappear of themselves, as do those remaining in mounts made without cells. The excess of balsam that flows over on the slide during the placing of the cover and closing of the cell, should be roughly wiped off with a wisp of tissue paper, so as to leave but a thin layer for the final cleaning, when the mount is hard and complete. Also, at this time, the cover should be carefully adjusted with the needles or forceps, so as to rest concentrically upon the surface of the cell, equally distant all around its circumference from the *outer* edge of the latter; and should be firmly pressed down upon the cell; and over the same. The cover, however, must never be pressed in its centre, or over the balsam. Being elastic, it readily bends downward under such pressure, driving out a portion of the balsam over the edge of the cell: the pressure being removed, the glass returns to its former level drawing in *air* to replace the expelled balsam, and thus compelling us to do our work all over again. The slide should now be set aside, in a warm but not hot place, and allowed to thoroughly harden, when the superfluous balsam may be scraped off with a dull knife blade, and the final cleaning made with a rag moistened with benzole, or better — chloroform; after which, soap and water will restore the pristine polish of the glass. The *finish* must be left to the taste of the worker. If all the proceedings, thus far, have been neatly and carefully executed, the slide will present an exceedingly neat appearance, without any ring of cement being run upon the edges of the cell, but it will look still better if finished with white zinc cement, and a narrow ring of color, as described in my first paper, care being taken to coat the edge of the cover, first, with shellac, or some other cement, which is insoluble in balsam or benzole.

These directions for mounting in balsam with cells of varying depths, and made of sheet wax, are identically the same as though we were using those of glass, metal, or other substance. Further, if our specimen (instead of being dry, as were the supposed foraminifera), has been carried through the preliminary stages of alcohol and oil of cloves, it may be transferred directly from the latter, or from benzole, care being taken merely to drain off all superfluity of either, and to avoid air bubbles in the immersion. Whole insects, large as blow-flies, may be thus mounted, without flattening or alteration in shape. Directions for so doing, and for rendering them sufficiently transparent, may be given in a future paper.

Even the simple wax cell may be beyond the immediate reach of many workers, who may still find a necessity for employing some substitute to prevent the crushing of a delicate object, or to hold the cover parallel over a specimen too thick for the ordinary mode of balsam mounting. For such, the following very simple contrivance can be made to answer a most useful purpose, and at the same time make a very neat appearance. Prepare a number of slips of paper and card-board of various thicknesses, by gumming upon one side. They may be placed in a convenient box for use when required. Selecting a slip slightly thicker than the object we desire to mount, we will proceed to cut four minute squares from it: then placing a cleansed slide upon our mounting plate, we will stick these squares thereon, at equal distances from the center. These distances will, of course, be regulated by the size of our object and the diameter of the covering glass, as the latter must cover all the squares when laid in position. The gum is to be moistened, of course, to attach the squares to the slide, and when they are in proper position, the whole must be warmed sufficiently to drive off *all* moisture, which would otherwise cause a milky appearance in the balsam. A drop of the latter having been placed in the centre of the slide and the specimen properly arranged therein, a cover is to be warmed and placed upon it according to former directions, pressing the same down gently with the forceps until it rests equally upon all four of the squares of paper or card-board. Should the object be decentered during this operation, it may be pushed back into position by a flattened needle introduced between the slide and the cover. This useful little tool can be readily made by drawing the temper from a large needle and hammering it out flat upon an anvil or any smooth hard surface of iron or steel; after which one end may be inserted into a match by way of handle. The needle should always be warmed over the lamp before thrusting it beneath the covering glass, to avoid the formation of air bubbles. If the balsam first applied be insufficient to fill out perfectly to the edges of the cover, more may be added by means of the glass rod. It will flow under the cover by capillary attraction and fill up evenly all around, if the operation be carefully performed. Then come the final operations of setting away to harden (always in a flat position), and the ringing with cement, or not, as the worker's fancy dictates. A mount made in this manner is very simple and easy, requires no outlay for even the materials for the cell, and can be made quite neat and handsome in appearance, as this faithful reproduction of one will testify to the reader's eye.

The mounting of diatoms in balsam is probably familiar to all of my readers, and yet, I feel impelled to give a few hints on this branch, albeit at the risk of telling what every one already knows full well. I do not propose to touch upon the cleaning of these at present; but, presuming all this preparatory work to have been already well done, I will briefly tell how to mount them in an easy and expeditious way.

For this purpose we shall require, in addition to the tools and materials already mentioned, a glass tube about six inches long with a bore of say $\frac{3}{16}$ of an inch, a homœopathic vial, or still better one made from glass tubing, and a brass table, larger and heavier than that shown in our first paper, with spirit lamp. Having these ready, with the cleaned diatoms in the stock bottle, and our balsam (pure), slips, circular covers, and mounting plate, let us proceed to work. Shake the stock-bottle carefully until its contents are evenly distributed throughout, then with the glass tube take up two or three dips and transfer to the small vial, which fill with distilled or pure filtered water, and allow the diatoms to settle to the bottom, when the fluid must be carefully poured off, and the vial again filled with water. The object of this is to get rid of almost every trace of the alcohol, which contained the diatoms in the stock bottle, and which, if not removed, would prevent them from being evenly distributed over the covers. I say *covers*, for diatoms should always be mounted upon these, and not upon the slides. Now, place upon the table as many covers as you desire to mount, separated some little distance from each other, and with the glass tube drop a portion of the contents of the small vial of diatoms in water upon each. The vial should be gently shaken between each dip, to distribute the diatoms evenly, and the amount of fluid placed on each cover should be sufficient to extend to the edge, and assume a convex form over it. The diatoms will distribute themselves evenly over the whole surface, care being taken not to disturb them by any jarring of the table. The dip is to be made from the vial by thrusting the tube down nearly to the bottom, and closing the top with the forefinger: the contents are deposited upon each cover by gently raising the finger and allowing the air to enter. A little practice is necessary to perform this successfully always. Our covers now being all ready, with watery burden covering the tiny diatoms, we light our spirit lamp and carefully apply its flame beneath the table, moving it back and forth and occasionally withdrawing it altogether, our object being to slowly evaporate the water without causing it to boil, or in any way disturb the diatoms. As soon as the evaporation is complete, the lamp may be left untouched and the table heated to the highest degree possible, in order to insure a thorough drying of the diatoms, which are now ready for the final mounting.

Place a slide upon the mounting plate and a minute drop of *pure* balsam in its centre, and, grasping one of the hot covers with the forceps, turn it over upon the balsam, which will quickly spread out to its edges, and the mounting is complete: done as quickly as I can tell of it. If desired, the slide may be heated over the lamp until all the turpentine is driven off, when, upon cooling, the cover will be found firmly fixed; but the preferable way is to set it aside for hardening in the usual manner. The smallest possible quantity of balsam should be used, and no ringing or other finish is necessary.

And now, having given hints as to various modes of making balsam mounts, I must bring this second installment of my talks to an end; having reached the limit of my space, without touching upon any other methods of mounting, as was my intention, and which must be left to another time.

It might be inferred, from the length at which I have dwelt upon balsam and its uses, that I hold it in supreme regard as a mounting medium. But such is by no means the case. Whilst it is invaluable as a preservative--and mountings made in it are probably nearer to being absolutely *permanent* than any other--it possesses many defects, which render it most unsuitable for mounting many delicate tissues which are so frequently placed in it, thereby becoming almost invisible. There are several aqueous fluids, which are far better suited to showing well the structure and beauties of innumerable objects, and these will be treated of in future papers. But their successful use, and the preparation and finishing of a permanent cell, containing them, requires a degree of skill to which the student can only attain by persistent efforts, and many failures; I have, therefore, thought it best to ground him well in the easier methods of balsam mountings, before proceeding to the higher plains. In the next paper, therefore, I propose to treat of dry mounts, many of the preliminary processes of which are identical with those required for fluids, and which will serve to lead up to the latter. A worker who can make his balsam mounts neatly and without failures, and, consequently, without saying or *thinking* naughty words, has certainly passed over the roughest portion of his journey, and may look with confidence for a continuously easier road, no matter in how many different directions it may lead him.

---

THE STENCHES FROM HUNTER'S POINT, L. I., are not so great as formerly. The State Board of Health, with commendable zeal, has succeeded in abating the nuisances connected with several of the manufactories of manure in that locality. There are, however, other establishments which persist in their objectionable methods, and will probably, in due time, be held to legal accountability.

